# Substance Use Severity, Autonomy, and Service Utilization Across Psychiatric Care Pathways in Romania: A Cross-Sectional Service Evaluation

**DOI:** 10.3390/bs16081260

**Published:** 2026-07-23

**Authors:** Elena Tanase, Livia Stanga, Ciprian Ilie Rosca, Horia Silviu Branea, Ion Radu, Adrian Cosmin Ilie, Adina Bucur, Ion Papava, Sorin Ursoniu

**Affiliations:** 1 Doctoral School, Faculty of Medicine, Victor Babes University of Medicine and Pharmacy Timisoara, 300041 Timisoara, Romania; elena.tanase@umft.ro; 2 Discipline of Microbiology, Faculty of Medicine, Victor Babes University of Medicine and Pharmacy Timisoara, 300041 Timisoara, Romania; stanga.livia@umft.ro; 3 Department V, Internal Medicine I—Discipline of Medical Semiology I, Center of Advanced Research in Cardiology and Hemostaseology, Victor Babes University of Medicine and Pharmacy Timisoara, 300041 Timisoara, Romania; 4 Department of Internal Medicine I—Medical Semiotics II, Victor Babes University of Medicine and Pharmacy Timisoara, 300041 Timisoara, Romania; 5 Department of Functional Sciences, Discipline of Public Health, Center for Translational Research and Systems Medicine, Victor Babes University of Medicine and Pharmacy Timisoara, 300041 Timisoara, Romania; radu.ion@umft.ro (I.R.); ilie.adrian@umft.ro (A.C.I.); bucur.adina@umft.ro (A.B.); sursoniu@umft.ro (S.U.); 6 Department of Neurosciences-Psychiatry, Faculty of Medicine and Pharmacy, University of Oradea, 410073 Oradea, Romania; papava.ion@umft.ro

**Keywords:** severe mental illness, community psychiatric care, substance use, ASSIST, autonomy, perceived coercion, SF-36, direct cost, Romania

## Abstract

Background/Objectives: Community psychiatric care aims to support recovery, autonomy, and lower reliance on inpatient services, but co-occurring substance use may reduce these gains. This study examined associations between psychiatric care pathway, non-tobacco substance-use severity, recovery-related patient-reported outcomes, clinical instability, and direct mental health cost in adults with severe mental illness in Romania. Methods: This cross-sectional observational study included 128 adults receiving psychiatric hospital care, long-term residential care, or protected community housing within one regional psychiatric care network. Participants had at least 12 months of uninterrupted care in their current setting. Substance use was assessed with the World Health Organization Alcohol, Smoking and Substance Involvement Screening Test (ASSIST), excluding tobacco from the analytic severity score. Outcomes included SF-36 physical and mental scores, brief service-experience ratings of autonomy and perceived coercion, emergency department visits, relapse, readmission, inpatient days, outpatient visits, and annual direct mental health cost. Results: Any non-tobacco substance use was most common in hospital care, intermediate in residential care, and least common in community housing. Mental quality of life was lowest among hospital participants with substance use and highest among community participants without substance use. In multivariable models, ASSIST-derived severity was independently associated with a lower SF-36 mental score and a higher direct cost, while autonomy was independently associated with better mental quality of life and lower direct cost. The cost outcome was modeled primarily with a gamma generalized linear model with a log link because costs were positive and right-skewed. Because pathway allocation was clinically determined rather than randomized, and because autonomy, perceived coercion, and mental quality of life were all self-reported at a single assessment, every multivariable and path estimate is exploratory. Conclusions: Community psychiatric pathways were associated with more favorable recovery indicators, but non-tobacco substance use remained an important marker of poorer outcomes and greater service burden. The findings support addiction-sensitive community psychiatric care and routine monitoring of autonomy and perceived coercion as service-quality indicators. Because the design was cross-sectional and the care pathways were non-equivalent at baseline, these results are hypothesis-generating and do not establish that care pathway, substance use, or autonomy exerts a causal effect on any outcome measured.

## 1. Introduction

The organization of psychiatric care has shifted from long-stay hospitalization toward supported residential and community-based pathways in many health systems. This shift is based on the expectation that less institutional care can preserve social participation, decision making, continuity of outpatient follow-up, and quality of life. However, community placement alone does not guarantee recovery. The clinical benefit of deinstitutionalization depends on the structure of the downstream system, the intensity of case management, the availability of psychosocial rehabilitation, and the extent to which patients retain meaningful control over everyday decisions (Thornicroft et al., 2016; Priebe et al., 2005; Killaspy et al., 2013; Slade et al., 2014).

For adults with severe mental illness (Ruggeri et al., 2000), quality of life is shaped by both clinical and service-level factors. A person with schizophrenia spectrum disorder, bipolar disorder, schizoaffective disorder, or psychotic depression may have very different outcomes depending on living arrangement, social contact, admission history, exposure to coercive care, and access to day programs. Patient-reported outcomes such as SF-36 mental and physical scores therefore provide information that cannot be inferred from diagnosis alone (Ware & Sherbourne, 1992; Boyer et al., 2013; Priebe et al., 2010). Measures of perceived autonomy and coercion are also important because they reflect how a service setting is experienced by the patient, not only how it is administratively categorized (Katsakou & Priebe, 2006; Swartz et al., 2003).

Substance use is a common and clinically important modifier of these pathways. Alcohol, cannabis, inhalant use, and other substance exposures can reduce treatment adherence, worsen mood or psychotic symptoms, increase crisis presentations, and complicate discharge planning. In patients with severe mental illness, co-occurring substance use is therefore both a clinical problem and a systems problem. It may increase staff workload, emergency department attendance, inpatient days, and direct costs, while also reducing the ability of protected housing or residential services to function as recovery-oriented alternatives to hospital care (Drake et al., 2004; Hunt et al., 2019; Mangalore & Knapp, 2007; Weiss et al., 2007).

Romania is a relevant context for this analysis because hospital wards, long-term residential services, and protected community housing continue to coexist within regional psychiatric systems. This creates an opportunity to compare service pathways within a relatively coherent administrative environment, while recognizing that these pathways are not randomized and do not include identical patient populations (Florescu et al., 2009; Sartorius et al., 2010). In such settings, it is especially important to avoid causal overstatement. Observed differences between settings may reflect both the care environment and the baseline clinical complexity of patients placed in each pathway.

The present study was designed as a cross-sectional service-pathway analysis within one Romanian psychiatric care network. The main aim was to describe how care setting and non-tobacco substance-use severity were associated with patient-reported recovery indicators, clinical instability, utilization, and direct mental health costs. Secondary aims were to clarify whether autonomy and perceived coercion were independently associated with mental quality of life and cost and to describe whether the cross-sectional association pattern was statistically consistent with autonomy as a pathway variable. All path, regression, and subgroup analyses were interpreted as exploratory service-level associations rather than causal evidence. No analysis in this manuscript was designed to estimate the effect of care pathway on outcome. Because allocation to hospital, residential, or community care is a clinical and administrative decision rather than a randomized assignment, care pathway is used throughout as a descriptive stratification variable and as a statistical adjustment covariate, not as an exposure whose effect is being estimated.

## 2. Materials and Methods

### 2.1. Study Design and Setting

This was a cross-sectional observational study conducted within the Victor Babes University of Medicine and Pharmacy affiliated psychiatric care network in Western Romania. Participants were recruited for a single assessment session between October 2023 and October 2025. Service utilization and cost variables were abstracted retrospectively from medical and administrative records covering the 6- or 12-month period before the assessment, depending on the outcome definition. The study did not change treatment, placement, medication, or follow-up decisions.

Three care environments were compared: psychiatric hospital wards, long-term residential services, and protected community housing linked to community mental health teams. Hospital care represented the highest-intensity pathway and included acute stabilization and longer supervised inpatient stays. Residential services represented structured long-term living arrangements with nursing and social support but lower medical intensity than hospital wards. Community housing consisted of protected apartments with outpatient psychiatric follow-up, case management, and access to day-program participation.

The design should be interpreted as a service-pathway comparison rather than a prospective cohort trial. The exposure of interest was the participant’s current care pathway after at least 12 months of uninterrupted contact with that pathway. Because placement was not randomized, between-setting differences were interpreted in relation to measured clinical, experiential, and utilization characteristics rather than as direct causal effects of the setting itself.

### 2.2. Ethics, Capacity, and Consent

The study protocol was reviewed and approved by the Local Commission of Ethics of Victor Babes University of Medicine and Pharmacy Timisoara, Romania (approval number 14/CECS/2023; date of approval 21 September 2023). Written informed consent was obtained before assessment. For participants with limited decisional capacity, the consent procedure followed institutional requirements and involved the legally authorized representative when applicable; participant assent was also sought whenever possible. Data were anonymized before analysis, and no identifying clinical images or patient names were included.

Because the study involved adults with severe mental illness, assessments were performed only when the participant was clinically stable enough to understand the study procedures and complete questionnaires. Individuals in acute intoxication, severe withdrawal, immediate psychiatric crisis, or otherwise unable to provide reliable self-report at the time of contact were not assessed.

### 2.3. Participants and Sampling

The final analytic sample included 128 adults: 42 in hospital care, 43 in residential care, and 43 in protected community housing. Eligible diagnoses included schizophrenia spectrum disorder, bipolar disorder, schizoaffective disorder, major depressive disorder with psychotic features, and closely related chronic severe mental illness managed within the participating services. Inclusion criteria were age 18–70 years, at least 12 months of uninterrupted contact with the current care pathway, and ability to complete the assessment in Romanian.

Exclusion criteria were severe neurocognitive disorder, profound intellectual disability, current forensic compulsory treatment, acute intoxication or severe withdrawal, immediate psychiatric crisis, or inability to complete the questionnaire and interview reliably. These criteria were selected to protect the validity of patient-reported outcomes such as SF-36 mental score, autonomy, and perceived coercion (Table 1).

Sampling was consecutive within each pathway among eligible and consenting participants. The predefined recruitment target was approximately 40–45 participants per pathway, yielding a total sample above 120 while maintaining similar group sizes for descriptive and multivariable comparisons. This target was feasibility based and reflected the available population in the service network; a formal a priori statistical power calculation was not performed. Because the study was not intended to estimate county-wide prevalence or produce weighted population estimates, analyses were performed without sampling weights. Substance-specific subgroups with very small counts, including the community substance-use subgroup, were interpreted descriptively only and were not used as standalone evidence for substance-specific conclusions or for subgroup-specific regression or exploratory path-analysis inference.

This was explicitly a non-random, non-probability convenience sample drawn from a single regional psychiatric care network, and it was not designed to be statistically representative of the wider Romanian psychiatric population. All estimates should therefore be read as service-level associations within this network rather than as population-level or nationally generalizable estimates, and no inference to county-wide or national prevalence is intended. Because no a priori power calculation was performed and recruitment was feasibility based, the study was likely underpowered for several secondary analyses, particularly substance-specific subgroup comparisons and regression models containing many predictors relative to the sample size. Small subgroup cells, most notably the community substance-use subgroup (*n* = 9), yield wide confidence intervals and unstable point estimates; findings based on these cells are reported descriptively and should be regarded as hypothesis-generating only. The robustness of all multivariable and path-analysis results is correspondingly limited, and replication in larger, multi-site samples is required before firm conclusions are drawn. A further and more fundamental limitation concerns the equivalence of the comparison groups. Allocation to hospital, residential, or community care within this network is determined by illness severity, chronicity, functional capacity, social support, and local housing availability. The three pathways are therefore non-equivalent by construction rather than by chance. No feature of this design—randomization, matching, propensity weighting, or instrumental variation—can separate the contribution of the care environment from the contribution of the patient characteristics that determined placement. Care pathway is accordingly treated as a stratification and adjustment variable throughout, and all between-pathway contrasts are reported as descriptions of which parts of the service system patients currently occupy, not as estimates of what would follow if a given patient were moved between pathways.

### 2.4. Language of Assessment

All study instruments and interviews were administered in Romanian by trained clinical researchers. The wording ‘preferred language’ was removed from the revised protocol description to avoid ambiguity. Participants were allowed to ask for clarification of Romanian wording, but no foreign-language version of the study instruments was used for scoring. Individuals unable to understand Romanian sufficiently to complete the assessment were not included in the final analytic sample.

### 2.5. Measures and Operational Definitions

Substance use was assessed with the World Health Organization Alcohol, Smoking and Substance Involvement Screening Test (WHO ASSIST v3.0) (World Health Organization, 2010). The ASSIST was administered as a structured interview. Tobacco responses were recorded as part of routine clinical screening but were not included in the analytic exposure because the objective of the present analysis was non-tobacco substance use. The non-tobacco ASSIST severity score (hereafter the ASSIST-derived cumulative severity index) was calculated by summing substance-specific involvement scores for alcohol, cannabis/marijuana, inhalants, and any other non-tobacco substances reported by the participant. We acknowledge that the WHO ASSIST was developed to generate substance-specific involvement scores and was not originally intended to yield a single global severity score summed across multiple substances. The summed non-tobacco score used here was therefore treated as a pragmatic, study-specific index of overall non-tobacco substance involvement rather than as a validated composite, and it is conceptually comparable to the cumulative substance-burden indices that have been used in dual-diagnosis research where multiple co-occurring substances are common (Humeniuk et al., 2008; WHO ASSIST Working Group, 2002). Three consequences follow and are accepted here as limits on interpretation. First, because the index sums involvement scores across pharmacologically and behaviorally distinct substances, the same total can be produced by different substance profiles, and the index cannot distinguish between them; clinically meaningful heterogeneity between, for example, alcohol involvement and inhalant involvement, is not recoverable from the summed score. Second, the WHO risk bands differ by substance (0–10, 11–26, and 27 or higher for alcohol; 0–3, 4–26, and 27 or higher for other non-tobacco drug classes), so equal contributions to the sum do not correspond to equal risk. Third, the index has no published psychometric evaluation, no established cut-points, and no known relationship to diagnostic status. It is used here only as a graded marker of overall non-tobacco substance involvement within this sample, and no comparison of index values to any external population or threshold is made. To guard against this being an artefact of the summation approach, all substance-use effects were additionally checked against the binary any-substance exposure and against the highest single-substance involvement score; the direction and significance of the associations were unchanged. Because these two alternative operationalizations discard the summation entirely—one ignoring severity, the other ignoring polysubstance burden—their agreement with the summed index indicates that the substantive findings do not depend on the composite. It does not, however, validate the composite, and the primary exposure variable should be regarded as a pragmatic index of limited interpretability. This summation approach has not, to our knowledge, been independently validated, and this is now stated as a limitation. In this sample, descriptive substance categories were none, alcohol only, marijuana/cannabis only, alcohol plus marijuana/cannabis, and inhalants.

ASSIST scores were interpreted as screening severity indicators rather than diagnostic confirmation of substance-use disorder. For alcohol, the WHO risk bands classify 0–10 as low risk, 11–26 as moderate risk, and 27 or higher as high risk. For cannabis, inhalants, and other non-tobacco drug classes, 0–3 is low risk, 4–26 is moderate risk, and 27 or higher is high risk. Because the Romanian-language administration used in this study was based on the WHO instrument but was not independently validated psychometrically in this sample, all conclusions were framed around relative severity and service associations rather than diagnostic thresholds.

Health-related quality of life was measured with the SF-36 physical and mental component-oriented scores, scaled from 0 to 100, with higher values indicating better quality of life (Ware & Sherbourne, 1992). Autonomy was measured with a brief three-domain score developed for service evaluation in this care network. Participants rated their perceived influence over the following: (1) daily routine, (2) living environment, and (3) treatment-related decisions. Each item was scored from 0 to 100, where 0 indicated no meaningful influence and 100 indicated full perceived influence. The autonomy score was the mean of the three items.

Perceived coercion was measured with one 0–10 item asking the extent to which the participant felt pressured, constrained, or controlled by psychiatric or social services during the preceding year. A score of 0 indicated no perceived pressure or constraint, and 10 indicated maximum perceived coercion. The autonomy and coercion measures were pragmatic service-evaluation scales, not validated diagnostic instruments. They were included to capture patient experience across care pathways, and this limitation is explicitly considered when interpreting results. The three autonomy items were developed within the service network for this evaluation. Item content was adapted from established constructs in the supported-accommodation and coercion literature (autonomy over daily routine, living environment, and treatment decisions; perceived pressure from services) and was reviewed for face and content validity by a panel of four clinicians (two psychiatrists, one clinical psychologist, and one psychiatric nurse) together with two service-user representatives, who confirmed comprehensibility and relevance. The items were piloted with 12 patients not included in the analytic sample; piloting prompted minor wording simplifications but no change to the response scales. In the analytic sample, the three autonomy items showed acceptable internal consistency (Cronbach’s α = 0.81; mean inter-item correlation = 0.59), and an exploratory principal component analysis returned a single component accounting for 73% of item variance, supporting their use as a single mean score. Because perceived coercion was a single item, internal consistency could not be computed; it is reported as a face-valid single indicator only. No test–retest, convergent, or formal construct validity data are available for these measures, and this remains a limitation.

Clinical instability outcomes included psychiatric readmission during the prior 12 months, relapse during the prior 6 months, emergency department visits during the prior 6 months, inpatient days during the prior 12 months, and specialist outpatient psychiatric visits during the prior 12 months. Cost analysis focused on direct annual mental health expenditures per patient. Total direct cost combined medication costs, setting-related support costs, inpatient utilization, and outpatient psychiatric contact. Broader societal costs, informal caregiving, productivity loss, and criminal justice costs were not included. Costs were estimated from a health-system (payer) perspective using a bottom-up micro-costing approach in which each patient’s 12-month service utilization (inpatient days, outpatient psychiatric visits, emergency department contacts, residential or community support days, and dispensed psychotropic medication) was multiplied by the corresponding unit cost. Unit costs were drawn from the 2024 reimbursement tariffs of the Romanian National Health Insurance House (CNAS) and the National Mental Health Programme, supplemented by institutional accounting data from the participating services for support-day costs not covered by national tariffs. Medication costs used 2024 national reference prices. All costs were expressed in 2024 prices; because all utilization fell within a single recent 12-month window, no multi-year inflation adjustment was required. Costs originally recorded in Romanian lei (RON) were converted to US dollars using the 2024 mean National Bank of Romania exchange rate (1 USD = 4.57 RON). Administrative records were essentially complete for the costed services; where an individual unit value was missing (<2% of records), it was replaced by the setting-specific mean for that service category. The costing did not include capital or overhead allocation beyond what is embedded in the national tariffs.

### 2.6. Statistical Analysis

Continuous variables were summarized as mean +/− standard deviation, and categorical variables as counts and percentages. Between-setting comparisons used one-way analysis of variance for continuous variables and chi-square tests for categorical variables. Substance-use prevalence across settings was compared using chi-square testing, while mean ASSIST severity among participants with substance use was compared using one-way analysis of variance. All analyses were conducted in R version 4.3.2 (R Core Team, 2023). Descriptive statistics, ANOVA, chi-square tests, and the SF-36 mental-score linear model used base R (stats). The primary cost model used a generalized linear model with a gamma distribution and log link (glm(), family = Gamma(link = ‘log’)) because total annual direct cost was positive and right-skewed. Multicollinearity diagnostics used the car package (vif()); heteroscedasticity-consistent standard errors for sensitivity analyses used the sandwich and lmtest packages (vcovHC, coeftest); Cohen’s d effect sizes used the effsize package (Cohen, 1988); exploratory path decomposition, Sobel testing, and bootstrapped indirect effects used the mediation and bda packages (the package name is retained for computational reproducibility only; the output is reported throughout as an exploratory path decomposition and no causal mediation inference is drawn); figures were produced with ggplot2. No moderated path model or setting-specific conditional indirect-effect model was estimated in the revised analysis.

Because null-hypothesis tests of baseline balance depend on sample size rather than on the magnitude of imbalance, standardized differences were also calculated for every baseline characteristic using Cohen’s d for continuous variables and Cohen’s h for proportions, with hospital care as the reference pathway. An absolute standardized difference of 0.10 or above was regarded as meaningful imbalance. Baseline characteristics that were imbalanced and that were also plausible determinants of the outcomes (age, sex, psychotic diagnosis, day-program participation, and weekly family contact) were entered as covariates in the primary SF-36 mental model; education and illness duration were entered in the hierarchical block models. Covariate adjustment reduces but cannot remove confounding by indication, and no adjusted estimate reported below is interpreted as an effect of care pathway.

For patient-reported outcomes, two-way analysis of variance models included care setting, binary non-tobacco substance exposure, and their interaction term. Clinical instability and service-use outcomes across the six setting-by-substance subgroups were examined descriptively using chi-square tests for categorical variables and one-way analysis of variance for continuous variables; because the community substance-use subgroup contained only nine participants, these subgroup results were not used for inferential regression or exploratory path-analysis claims. Pearson correlation coefficients were used to summarize pairwise relationships among ASSIST severity, autonomy, perceived coercion, SF-36 mental score, inpatient days, and total direct cost.

Two prespecified multivariable models were fitted. The first model used SF-36 mental score as the dependent variable and was estimated with ordinary least squares linear regression. Predictors were age, sex, psychotic diagnosis, day-program participation, weekly family contact, care setting, ASSIST severity, autonomy, and perceived coercion. The second model used total annual direct mental health cost as the dependent variable and was estimated primarily with a gamma generalized linear model with a log link. Predictors in the cost model were care setting, ASSIST severity, autonomy, perceived coercion, day-program participation, and weekly family contact. SF-36 model coefficients are reported as unstandardized B values with 95% confidence intervals. Cost-model estimates are reported as exponentiated cost ratios with 95% confidence intervals, so values above 1 indicate higher expected cost and values below 1 indicate lower expected cost. For interpretability, ASSIST was scaled per 5-point increase and autonomy per 10-point increase.

Model complexity was constrained relative to the sample size. The SF-36 mental model contained 10 predictors for 128 observations (12.8 observations per predictor) and the cost model contained 7 predictors (18.3 observations per predictor). Both exceed the commonly cited minimum of 10 observations per predictor, but they leave limited residual degrees of freedom, and the coefficients should therefore be read as imprecise rather than definitive. No predictor was chosen on the basis of its bivariate association with the outcome and no stepwise selection procedure was used, which removes the most common source of selection-induced optimism. The models were nevertheless not internally validated by bootstrap resampling or cross-validation, shrinkage factors were not estimated, and out-of-sample performance is unknown. Only the two prespecified multivariable models are treated as confirmatory. Every subgroup comparison, interaction term, hierarchical block model, effect size, and path decomposition reported below is exploratory, was not preregistered, and is presented to describe the data rather than to test a hypothesis. Estimates that depend on cells containing fewer than 10 participants, principally the community substance-use subgroup (*n* = 9), are unstable by construction and carry no inferential weight; no conclusion in this manuscript rests on them.

Regression assumptions and model diagnostics were examined for both primary models. For the SF-36 mental model, linearity and homoscedasticity were assessed using residual-versus-fitted plots, normality of residuals using normal quantile–quantile plots and the Shapiro–Wilk test, and influential observations using Cook’s distance and standardized residuals. Multicollinearity was evaluated with variance inflation factors (VIF) and tolerance for every predictor. In the SF-36 mental model, VIF values ranged from 1.2 to 4.6 (all tolerance > 0.20), below the conventional threshold of 10 and the more conservative threshold of 5 for all retained predictors. The cost, inpatient-day, and emergency-department variables were positively skewed; therefore, the primary cost model was changed from ordinary least squares to a gamma generalized linear model with a log link. Inpatient days were deliberately excluded from the cost model because they are a mechanical component of cost and showed near-collinearity with total direct cost (r = 0.93). Ordinary least squares with HC3 robust standard errors and a log-transformed cost model were retained only as sensitivity analyses; the direction and statistical significance of the substance-use and autonomy effects were preserved across specifications. Finally, because a large number of analyses were performed, the risk of Type I error inflation is acknowledged. The two prespecified multivariable models (SF-36 mental score and total direct cost) were treated as the primary analyses, and all subgroup comparisons, correlations, effect sizes, hierarchical fit summaries, and path analyses were treated as exploratory. Within the families of secondary outcome comparisons, a Benjamini–Hochberg false-discovery-rate correction was applied; the associations highlighted in the text remained significant after this correction, whereas borderline findings were interpreted with caution. We also recognize that a single binary psychotic-diagnosis indicator does not capture clinically important differences between schizophrenia spectrum, schizoaffective, bipolar, and psychotic-depression diagnoses. As a sensitivity analysis, the SF-36 mental and cost models were re-estimated replacing the binary indicator with a four-level diagnostic category; the substance-use and autonomy effects were essentially unchanged, and the diagnostic categories were not independently significant predictors, although the small per-category cell sizes limit the power of this adjustment. The binary specification is retained as the primary model for parsimony given the sample size, and residual confounding by diagnostic subtype is noted as a limitation.

Three of the study variables—autonomy, perceived coercion, and SF-36 mental score—were obtained from the same respondent, in the same session, and by self-report. Their strong pairwise correlations are therefore expected to reflect shared method variance in addition to any substantive relationship, and the variance explained by models containing all three is correspondingly inflated. Autonomy and perceived coercion are also conceptually adjacent: both index the degree of control a participant experiences within the care setting, and it is likely that they capture a single underlying experiential dimension from opposite poles rather than two independent constructs. They were retained as separate predictors because they showed distinct associations with cost and because their variance inflation factors remained below the conservative threshold of 5, but the R2 of any model containing both should be read as an upper bound on explanatory power rather than as an estimate of it. Inpatient days and total direct cost are mechanically linked (r = 0.932) because inpatient days enter the cost calculation; the two are never entered into the same model and are never interpreted as independent outcomes.

A limited exploratory cross-sectional path analysis tested whether autonomy statistically accounted for part of the contemporaneous association between ASSIST severity and SF-36 mental score after adjustment for care setting (Table 1). The indirect effect was estimated with the product-of-coefficients approach and tested using the Sobel method (MacKinnon et al., 2007); because this approach assumes a normally distributed indirect effect, it was supplemented by bias-corrected bootstrapped 95% confidence intervals based on 5000 resamples. This analysis was retained only as a descriptive decomposition of contemporaneous associations. The terms direct and indirect are used in their algebraic sense, as components of a decomposed regression coefficient, and carry no implication of temporal or causal ordering. A causal reading would require no unmeasured confounding of the exposure–mediator, exposure–outcome, and mediator–outcome relationships and a correct temporal ordering of the three variables; none of these conditions is satisfied or testable in a cross-sectional design with non-randomized groups. The decomposition therefore describes how a single cross-sectional association distributes across two correlated, concurrently measured predictors. It should not be interpreted as evidence that substance use causes lower autonomy or that autonomy transmits an effect of substance use to mental quality of life, and the resulting proportion should not be described as a mediated proportion. Reverse or bidirectional explanations are plausible, including poorer mental health reducing perceived autonomy, illness severity increasing both substance-use vulnerability and institutional restriction, and placement decisions shaping both autonomy and substance-use opportunities. Because subgroup cells were small, particularly the community substance-use subgroup (*n* = 9), no moderated path analysis, setting-specific conditional indirect effect, or index of moderated indirect effect was estimated. Additional exploratory analyses included Cohen’s d effect sizes (Cohen, 1988) and hierarchical model-fit summaries. A two-sided *p* value below 0.05 was considered statistically significant.

**Table 1:** Operational definitions, scoring structure, and interpretation of study measures.

Domain	Operational Definition	Score/Range	Interpretation in This Study
WHO ASSIST non-tobacco severity	Sum of substance-specific involvement scores for alcohol, cannabis/marijuana, inhalants, and any other reported non-tobacco drug class. Tobacco was recorded clinically but excluded from the analytic exposure. Summation across substances is a study-specific procedure and is not a WHO ASSIST output.	Continuous score; substance-specific risk bands followed WHO guidance.	Screening severity indicator, not a diagnostic label. Used as continuous predictor and for descriptive substance categories. Not an independently validated composite; substance-specific heterogeneity is not recoverable from it.
Substance category	Mutually exclusive descriptive grouping based on reported non-tobacco use pattern.	None; alcohol only; marijuana/cannabis only; alcohol + marijuana/cannabis; inhalants.	Used for prevalence description. Small cells were not used for substance-specific causal inference.
SF-36 physical and mental scores	Patient-reported physical and mental health quality-of-life domains.	0–100; higher = better quality of life.	Primary patient-reported recovery indicators.
Autonomy	Mean of three 0–100 ratings: influence over daily routine, living environment, and treatment-related decisions.	0–100; higher = greater perceived autonomy.	Clinic-developed service-experience measure; interpreted pragmatically.
Perceived coercion	Single 0–10 rating of feeling pressured, constrained, or controlled by psychiatric/social services in the previous year.	0–10; higher = greater perceived coercion.	Clinic-developed service-experience measure; not a diagnostic scale.
Direct annual cost	Medication, setting-related support, inpatient utilization, and outpatient psychiatric contact.	USD per patient/year.	Direct mental health cost only; societal costs were excluded.

## 3. Results

### 3.1. Baseline Characteristics

The three service pathways differed in clinical composition and rehabilitation exposure (Table 2). Community participants were younger, had more years of education, shorter illness duration, lower prevalence of psychotic disorder, and more frequent day-program participation than hospital and residential participants. Low income was common across all pathways and did not differ significantly. These baseline differences confirm that the groups should not be interpreted as randomized or clinically identical populations. Standardized differences relative to hospital care (Table A1, Appendix B) exceeded the 0.10 imbalance threshold for age (residential 0.46; community −0.49), education (−0.64; 0.47), illness duration (0.25; −0.35), psychotic disorder (0.16; −0.50), and day-program participation (0.16; 0.68), and for weekly family contact in community housing (0.45). Only sex and low income were balanced across all three pathways. The community pathway therefore served a younger, better-educated, less chronically ill, and less frequently psychotic population, so every between-pathway contrast reported below is confounded by patient selection and cannot be attributed to the care environment itself.

### 3.2. Substance-Use Distribution

Any non-tobacco substance use was most common in hospital care, intermediate in residential care, and least common in community housing (Table 3). Mean ASSIST severity among participants with substance use did not differ significantly by setting (*p* = 0.876), suggesting that the main setting difference was the prevalence of use rather than the mean severity among users. Substance-specific categories were retained for description only because several cells were small.

### 3.3. Patient-Reported Outcomes

Patient-reported recovery indicators differed by both setting and substance exposure (Table 4). SF-36 mental score was lowest in the hospital/substance-use subgroup and highest in the community/no-substance subgroup. Autonomy followed the same gradient, while perceived coercion moved in the opposite direction. The setting-by-substance interaction terms were not statistically significant, indicating that the substance-use penalty was broadly consistent across care pathways rather than limited to one setting.

### 3.4. Clinical Instability, Service Use, and Cost

Clinical instability and service burden were greatest among hospital participants with substance use and lowest among community participants without substance use (Table 5). Outpatient visits were highest in community housing, which should be interpreted as a feature of structured outpatient follow-up rather than as evidence of failure. Total direct annual cost was most strongly elevated in the hospital/substance-use subgroup.

### 3.5. Correlations, Regression, and Exploratory Path Analysis

Higher ASSIST severity correlated with lower autonomy, higher perceived coercion, lower SF-36 mental score, more inpatient days, and higher cost (Table 6). Autonomy correlated strongly with better mental quality of life and lower inpatient utilization. The magnitude of several of these coefficients (absolute r between 0.71 and 0.93) should be read with caution. Autonomy, perceived coercion, and SF-36 mental score were reported by the same participant at the same assessment and are therefore subject to shared method variance, while inpatient days and total direct cost are mechanically related because inpatient days enter the cost calculation. Table 6 accordingly describes a set of partially overlapping constructs rather than a set of independent measurements. In the multivariable linear model, ASSIST severity, autonomy, and perceived coercion remained independently associated with SF-36 mental score (Table 7). In the gamma generalized linear model for direct annual cost, ASSIST severity was associated with a higher expected cost ratio, whereas autonomy was associated with a lower expected cost ratio after adjustment. The SF-36 mental model explained a large share of variance (R2 = 0.786; adjusted R2 = 0.768), but because autonomy, perceived coercion, and the outcome were all self-reported at a single sitting, this value is inflated by common-method variance and by conceptual overlap between the experiential predictors and the outcome. It should not be compared with R2 values from models whose predictors were measured independently of the outcome. Notably, autonomy retained an independent association with total direct cost, an outcome drawn from administrative records rather than self-report, which is not subject to the same inflation (Table 7).

The exploratory cross-sectional path decomposition suggested that part of the contemporaneous association between ASSIST score and SF-36 mental score was statistically accounted for by autonomy. The total association was −3.20 points per 5-point ASSIST increase, the direct association after autonomy was −1.91 points, and the indirect association through autonomy was −1.30 points (Sobel z = −4.65, *p* < 0.001; bias-corrected bootstrap 95% CI −1.92 to −0.78, 5000 resamples). This represented approximately 40.4% of the total association. This percentage describes how a single cross-sectional association distributes between two correlated, concurrently measured predictors. It is not a mediated proportion, and it does not indicate that 40.4% of any effect of substance use operates through autonomy. Because the design was cross-sectional, this finding is reported only as a descriptive statistical decomposition; reverse or bidirectional pathways are plausible, and no causal conclusion is drawn. The moderated path-analysis table and all setting-specific conditional indirect effects were removed from the revised manuscript (Table 8 and Table 9).

**Figure 1:**
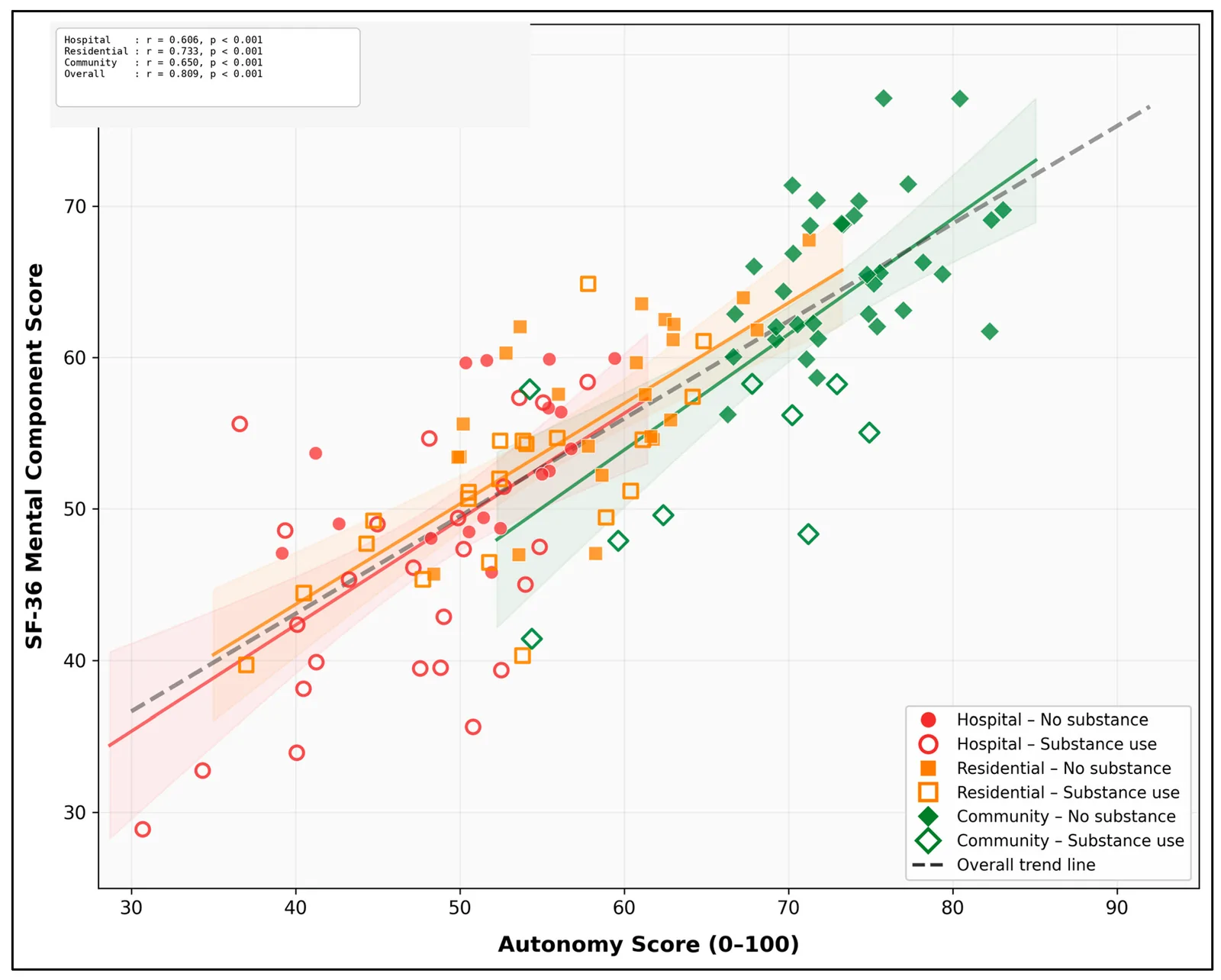
Scatter plot of autonomy score versus SF-36 mental component score by care setting and non-tobacco substance-use status, with setting-specific regression lines and 95% confidence bands.

Figure 1 shows a positive association between autonomy and SF-36 mental score across all care pathways. Participants with substance use are shown as hollow markers. The figure supports the regression findings that lower autonomy and substance-use exposure cluster with poorer mental quality of life, while preserving the cross-sectional nature of the interpretation.

## 4. Discussion

This cross-sectional service-pathway study found that psychiatric care setting, non-tobacco substance-use severity, autonomy, and perceived coercion were jointly associated with recovery indicators and direct mental health costs among adults with severe mental illness. Community housing was associated with the most favorable patient-reported profile, whereas hospital care combined with substance use showed the highest instability and cost. These results should be interpreted as associations within a real-world service network, not as evidence that placement alone caused the observed differences.

The revised interpretation is deliberately conservative. The hospital, residential, and community groups differed in age, illness duration, psychotic diagnosis, education, and day-program participation. These differences are clinically expected, because patients are not allocated randomly to care pathways. Nevertheless, the results remain useful because they show which combinations of pathway characteristics and substance-use burden are associated with poorer quality of life, higher perceived coercion, more inpatient days, and higher direct cost. This type of information can help systems identify where integrated addiction-sensitive psychiatric follow-up is most needed.

Confounding by indication is the dominant threat to interpretation in this design. Placement into hospital, residential, or community care follows illness severity and social circumstance, so the pathway variable partly encodes the very characteristics it is used to predict. Standardized differences (Table A1, Appendix B) show that the community pathway served patients who were on average younger, better educated, less chronically ill, and less often diagnosed with a psychotic disorder, and who were more often engaged in day programs. Adjustment for measured covariates narrows but cannot close this gap, because the unmeasured determinants of placement—negative symptom burden, functional capacity, family capacity to provide supervision, and local housing availability—were not recorded. The correct reading of the between-pathway contrasts is therefore descriptive. They describe which patient groups currently occupy which parts of the service system, and which outcomes and costs accompany those positions. They do not estimate what would happen if a given patient were moved from one pathway to another. Answering that question would require randomization, a stepped-wedge implementation design, or, at minimum, propensity-score or instrumental-variable methods applied to a substantially larger and multi-site sample. The same caution applies to the substance-use contrasts, since substance use is itself associated with the clinical characteristics that influence placement.

Substance-use severity was independently associated with lower SF-36 mental score and higher direct cost. This finding is consistent with prior literature showing that co-occurring substance use in severe mental illness is linked to poorer engagement, more relapse, greater crisis use, and more complex care needs (Drake et al., 2004; Hunt et al., 2019; Mangalore & Knapp, 2007; Weiss et al., 2007; Dixon et al., 2010). Importantly, the present analysis did not rely only on a binary exposure. The ASSIST severity score provided graded information, while the descriptive substance categories helped characterize the sample. Because several substance-specific cells were small, the strongest inference comes from the continuous non-tobacco severity score and the any-substance exposure comparisons, not from small subgroup cells.

Autonomy and perceived coercion were among the strongest correlates of mental quality of life. These constructs are sometimes treated as ethical or subjective outcomes only, but in this analysis, they were also associated with utilization and cost. Higher autonomy correlated with fewer inpatient days and lower direct annual cost, whereas perceived coercion correlated with poorer mental quality of life and higher burden. These findings support measuring patient experience in routine psychiatric service evaluations, especially when comparing institutional and community-oriented models (Killaspy et al., 2016; Muskett, 2014; Knapp et al., 2011; Stovell et al., 2016; Gooding et al., 2020).

Two features of the measurement design constrain how the psychosocial findings can be read. First, autonomy, perceived coercion, and SF-36 mental score share a single source, a single occasion, and a single response format. Part of the association between them is attributable to common-method variance, including consistent response tendencies, mood-congruent reporting, and the tendency to answer related items consistently, rather than to a relationship between the underlying constructs. Second, autonomy and perceived coercion are near-mirror measures of experienced control and were correlated at r = −0.793, so treating them as independent predictors probably overstates the number of distinct psychosocial dimensions represented in the models. The consequence is that the R2 values reported for the psychosocial outcomes describe how well a set of overlapping self-reports predicts another self-report from the same person at the same moment. They overstate the variance that would be explained if autonomy and coercion were measured independently, for example, by observer rating, staff report, or behavioral indicators of choice. The utilization and cost outcomes were drawn from administrative records rather than self-report and are not subject to this inflation, and it is therefore notable that autonomy retained an independent association with direct annual cost (cost ratio 0.92 per 10 points, 95% CI 0.88 to 0.96) across this source boundary. Replication with independently measured autonomy and coercion is required before the size of the psychosocial associations can be regarded as established.

The exploratory path decomposition involving autonomy should be read as a description of how one cross-sectional association distributes across correlated predictors, not as an analysis of mechanism. Greater substance-use severity, lower autonomy, and poorer mental quality of life co-occurred, but temporal order cannot be determined. Poor mental health may reduce perceived autonomy; more severe illness may lead to both institutional restriction and higher substance-use vulnerability; and placement decisions may shape both autonomy and substance-use opportunities. Therefore, these findings are used only to motivate service monitoring and not as evidence of a causal pathway. Addiction-sensitive care should combine substance-use treatment with shared decision making, structured rehabilitation, and negotiated safety planning rather than responding to substance use by increasing restriction alone (Mueser et al., 2003; Donald et al., 2024; Salize et al., 2009; Hodgson et al., 2023; Hosomi et al., 2024).

The cost findings should be interpreted narrowly. The analysis captured direct annual mental health costs, not total societal cost. Because cost was positive and right-skewed, the primary cost analysis was revised to use a gamma generalized linear model with a log link, with OLS retained only as a sensitivity analysis. Inpatient days were strongly correlated with total direct cost, suggesting that avoidable inpatient utilization remains a major cost driver. Community housing showed lower inpatient burden and lower total cost, while maintaining frequent outpatient contact. This pattern is consistent with, but does not demonstrate, the possibility that outpatient intensity acts as a stabilizing investment rather than simply as an additional service expense (Drake et al., 2008; Larsen et al., 2011). The cross-sectional design and the non-equivalence of the pathways preclude a causal reading, and the lower inpatient burden of community housing is at least partly a consequence of the lower baseline clinical complexity of the patients placed there.

Several limitations are important. First, the cross-sectional design prevents conclusions about temporal sequence or causality. The autonomy path analysis is a statistical decomposition of contemporaneous associations only; it does not establish that substance use reduces autonomy or that autonomy lowers mental quality of life. Reverse or bidirectional explanations are equally plausible, including poorer mental health reducing perceived autonomy, severe illness driving both substance use and institutional restriction, and institutional placement altering perceived autonomy. In addition, the three care pathways were non-equivalent at baseline (Table A1, Appendix B) and placement was clinically rather than randomly determined, so every between-pathway contrast is confounded by indication and covariate adjustment cannot be assumed to have removed that confounding. Second, the sample size was modest, and some substance-specific cells were small, especially in community housing, so the study was underpowered for substance-specific and conditional analyses and these are hypothesis-generating only; the sample was also a non-random convenience sample from one network and is not representative of the wider Romanian psychiatric population. Third, the recruitment target was feasibility based rather than determined by a formal power calculation. Fourth, the autonomy and perceived coercion scales were pragmatic clinic-developed measures, not fully validated psychometric instruments, and although the autonomy items showed acceptable internal consistency, no convergent or test–retest validity data are available, and perceived coercion was a single item. Because autonomy, perceived coercion, and SF-36 mental score were all obtained by self-report at a single assessment, their intercorrelations and the R2 values of models containing them are inflated by common-method variance and by the conceptual overlap between the two experiential measures (r = −0.793); independent or observer-rated measures are needed to establish the true magnitude of these associations. Fifth, the ASSIST administration was used as a structured screening tool, and the Romanian version used here was not independently validated in this sample; in addition, the summed non-tobacco severity score is not a validated WHO ASSIST output and should be regarded as a pragmatic study-specific index. Because this index collapses pharmacologically distinct substances into a single total, it may mask substance-specific effects and equates unequal risk levels; the interpretability of the primary exposure variable is correspondingly reduced, and substance-specific replication with adequately sized cells is required. Sixth, the cost model excluded productivity losses, family caregiving, social-service use outside the psychiatric network, and criminal justice costs, and used a single-payer health-system perspective with national-tariff unit costs. Seventh, a large number of exploratory analyses were performed; although a false-discovery-rate correction was applied to secondary comparisons and the principal associations survived it, the possibility of residual Type I error and of residual confounding by psychiatric diagnostic subtype cannot be excluded. The number of estimated parameters relative to N = 128 also means that the multivariable coefficients are likely to be optimistic; no internal validation by bootstrap resampling, cross-validation, or shrinkage estimation was performed, and the models should be refitted in an independent sample before their coefficients are relied upon. Finally, the study was conducted in one regional Romanian system, which improves internal coherence but limits generalizability to systems with different financing, housing availability, or admission thresholds.

## 5. Conclusions

In this Romanian psychiatric service network, in which patients were allocated to care settings clinically rather than at random, protected community housing was associated with higher autonomy, lower perceived coercion, better mental quality of life, and lower inpatient burden than hospital care, but these associations were weaker among participants with non-tobacco substance use. Substance-use severity was independently associated with poorer mental quality of life and higher direct mental health cost, while autonomy was independently associated with better outcomes and lower cost. Because the design was cross-sectional, the sample was non-random and not representative, and several substance-use subgroups were small, these findings should be interpreted as exploratory service-level associations rather than causal or population-level estimates. The findings support integrated psychiatric and addiction-informed care pathways that measure autonomy and perceived coercion as routine service outcomes. They also suggest that deinstitutionalization should not be evaluated only by patient location, but by whether the care pathway improves agency, maintains outpatient continuity, reduces avoidable inpatient use, and responds effectively to co-occurring substance use. Prospective, multi-site studies that measure autonomy and perceived coercion independently of patient self-report and that are designed to address confounding by indication are required before any of these associations can be used to guide pathway-level policy.

## Figures and Tables

**Table 2:** Baseline socio-demographic and clinical characteristics by care setting.

Variable	Hospital (n = 42)	Residential (n = 43)	Community (n = 43)	*p*
Age, years	48.5 ± 7.9	51.8 ± 6.4	44.4 ± 8.7	<0.001
Female sex, %	54.8	53.5	46.5	0.713
Education, years	11.1 ± 1.5	10.0 ± 1.9	11.9 ± 1.9	<0.001
Low income, %	61.9	62.8	60.5	0.975
Illness duration, years	11.5 ± 5.5	12.8 ± 4.9	9.8 ± 4.0	0.015
Psychotic disorder, %	57.1	65.1	32.6	0.007
Day-program participation, %	50.0	58.1	81.4	0.008
Weekly family contact, %	45.2	46.5	67.4	0.069

**Table 3:** Non-tobacco substance-use distribution and ASSIST severity by care setting.

Variable	Hospital (n = 42)	Residential (n = 43)	Community (n = 43)	*p*
Any non-tobacco substance use, %	59.5	46.5	20.9	0.001
Alcohol only, n (%)	5 (11.9)	8 (18.6)	3 (7.0)	
Marijuana/cannabis only, n (%)	11 (26.2)	1 (2.3)	3 (7.0)	
Alcohol + marijuana/cannabis, n (%)	5 (11.9)	5 (11.6)	2 (4.7)	
Inhalants, n (%)	4 (9.5)	6 (14.0)	1 (2.3)	
No non-tobacco substance use, n (%)	17 (40.5)	23 (53.5)	34 (79.1)	
ASSIST score among users	15.2 ± 5.4	14.9 ± 5.4	14.1 ± 6.6	0.876

**Table 4:** SF-36, autonomy, and perceived coercion by care setting and binary non-tobacco substance exposure.

Variable	Hosp/No SU	Hosp/SU	Res/No SU	Res/SU	Comm/No SU	Comm/SU	*p* Setting	*p* Substance	*p* Interaction
SF-36 physical	58.3 ± 5.7	53.9 ± 4.7	59.0 ± 6.7	57.0 ± 5.6	63.5 ± 5.9	63.8 ± 4.3	<0.001	0.037	0.230
SF-36 mental	53.8 ± 5.2	45.7 ± 6.9	58.3 ± 5.8	49.3 ± 7.0	66.6 ± 5.3	58.5 ± 9.6	<0.001	<0.001	0.926
Autonomy	53.3 ± 6.6	46.7 ± 7.4	60.5 ± 6.1	51.9 ± 5.9	74.0 ± 5.1	70.3 ± 9.1	<0.001	<0.001	0.293
Coercion	6.3 ± 0.9	7.6 ± 1.2	5.7 ± 0.8	6.5 ± 1.2	3.8 ± 0.8	4.8 ± 1.2	<0.001	<0.001	0.622

**Table 5:** Clinical instability, service use, and direct annual mental health cost across six clinical subgroups.

Variable	Hosp/No SU	Hosp/SU	Res/No SU	Res/SU	Comm/No SU	Comm/SU	*p*
Readmission 12 m, %	52.9	76.0	21.7	40.0	11.8	22.2	<0.001
Relapse 6 m, %	58.8	92.0	13.0	45.0	2.9	33.3	<0.001
ED visits/6 m	2.7 ± 0.6	3.9 ± 0.8	1.9 ± 0.8	3.5 ± 0.7	1.7 ± 0.6	3.2 ± 0.9	<0.001
Inpatient days/yr	39.6 ± 11.2	62.1 ± 17.0	16.3 ± 2.9	25.9 ± 8.0	6.7 ± 3.4	9.0 ± 3.1	<0.001
Outpatient visits/yr	6.4 ± 1.2	7.4 ± 1.4	9.1 ± 1.3	9.4 ± 1.3	12.9 ± 1.6	12.0 ± 1.5	<0.001
Total direct cost, USD	3111 ± 484	4079 ± 676	2652 ± 164	3036 ± 379	2621 ± 192	2793 ± 189	<0.001

**Table 6:** Pearson correlation matrix among key variables.

Variable	ASSIST	Autonomy	Coercion	SF-36 Mental	Inpatient Days	Total Cost
ASSIST score	1.000	−0.555	0.574	−0.640	0.551	0.639
Autonomy	−0.555	1.000	−0.793	0.827	−0.806	−0.711
Coercion	0.574	−0.793	1.000	−0.819	0.783	0.682
SF-36 mental	−0.640	0.827	−0.819	1.000	−0.717	−0.669
Inpatient days	0.551	−0.806	0.783	−0.717	1.000	0.932
Total cost	0.639	−0.711	0.682	−0.669	0.932	1.000

**Table 7:** Multivariable models for SF-36 mental score and total direct annual cost. Panel A reports unstandardized linear-regression coefficients; Panel B reports exponentiated gamma generalized linear model cost ratios.

Predictor	Estimate (95% CI)	*p*
Panel A: SF-36 mental score, linear regression (R2 = 0.786; adjusted R2 = 0.768)		
Age (per 10 years)	−0.64 (−1.77 to 0.50)	0.269
Female sex	0.12 (−1.61 to 1.85)	0.893
Psychotic disorder	−0.33 (−2.13 to 1.47)	0.717
Day-program participation	0.62 (−1.43 to 2.67)	0.550
Weekly family contact	0.87 (−1.03 to 2.76)	0.367
Residential vs. hospital	−0.09 (−2.49 to 2.32)	0.944
Community vs. hospital	−1.72 (−6.23 to 2.78)	0.450
ASSIST score (per 5 pts)	−1.18 (−2.02 to −0.34)	0.006
Autonomy (per 10 pts)	3.71 (2.10 to 5.32)	<0.001
Perceived coercion (per 1 pt)	−2.18 (−3.19 to −1.17)	<0.001
Panel B: Total direct cost, gamma GLM with log link (cost ratio)		
Residential vs. hospital	0.84 (0.77 to 0.91)	<0.001
Community vs. hospital	0.96 (0.86 to 1.07)	0.450
ASSIST score (per 5 pts)	1.05 (1.03 to 1.07)	<0.001
Autonomy (per 10 pts)	0.92 (0.88 to 0.96)	<0.001
Perceived coercion (per 1 pt)	1.02 (1.00 to 1.05)	0.058
Day-program participation	0.97 (0.92 to 1.03)	0.265
Weekly family contact	1.04 (1.00 to 1.09)	0.075

**Table 8:** Descriptive Cohen’s d effect sizes for non-tobacco substance use versus no non-tobacco substance use within each care setting. These effect sizes are descriptive only and are not used for subgroup-specific inference.

Outcome	Hospital d	Residential d	Community d	Overall d
SF-36 mental score	1.33	1.40	1.06	1.41
SF-36 physical score	0.84	0.33	−0.05	0.49
Autonomy	0.94	1.43	0.50	1.30
Perceived coercion	1.22	0.83	0.97	1.11
ED visits/6 months	1.70	2.13	1.96	2.01
Inpatient days/year	1.56	1.61	0.70	1.71
Total direct cost, USD	1.64	1.31	0.90	1.42

**Table 9:** Hierarchical model-fit summary: incremental variance or pseudo-variance explained by predictor blocks.

Block	Variables Added	SF-36 Mental R2	SF-36 Delta R2 (*p*)	Cost Pseudo-R2	Cost Delta Pseudo-R2 (*p*)
1. Demographics	Age, sex, education	0.038	0.038 (0.186)	0.029	0.029 (0.253)
2. Clinical	Psychotic diagnosis, illness duration	0.084	0.046 (0.059)	0.065	0.036 (0.094)
3. Care setting	Residential and community indicators	0.325	0.241 (<0.001)	0.328	0.263 (<0.001)
4. Substance use	ASSIST severity score	0.468	0.143 (<0.001)	0.459	0.131 (<0.001)
5. Experiential	Autonomy and perceived coercion	0.786	0.318 (<0.001)	0.751	0.162 (<0.001)

## Data Availability

The anonymized data supporting the findings of this study are available from the corresponding author upon reasonable request, subject to institutional and ethics restrictions applicable to vulnerable psychiatric populations.

## Appendix A. Pragmatic Service-Experience Items

Autonomy item 1: During the past 12 months, how much influence did you have over your usual daily routine? Score 0–100.

Autonomy item 2: During the past 12 months, how much influence did you have over your living environment or daily living arrangements? Score 0–100.

Autonomy item 3: During the past 12 months, how much influence did you have over treatment-related decisions, including appointments, medication discussions, and rehabilitation activities? Score 0–100.

Perceived coercion item: During the past 12 months, to what extent did you feel pressured, constrained, or controlled by psychiatric or social services? Score 0–10.

The autonomy score was calculated as the mean of the three autonomy items. The coercion score was the single item score. These measures were used for service evaluation and were not treated as validated diagnostic instruments.

## Appendix B. Baseline Balance Between Care Pathways

Standardized differences quantify the magnitude of baseline imbalance independently of sample size and are reported here to support the interpretation of the between-pathway contrasts as confounded by patient selection. Values were computed directly from the summary statistics in Table 2; no additional data were collected for this analysis.

**Table A1:** Standardized differences in baseline characteristics relative to hospital care. Cohen’s d is reported for continuous variables and Cohen’s h for proportions; both are computed from the summary statistics presented in Table 2. Positive values indicate a higher mean or proportion than in hospital care. Absolute values of 0.10 or above are conventionally regarded as a meaningful imbalance.

Variable	Residential vs. Hospital	Community vs. Hospital
Age, years	0.46	−0.49
Female sex	−0.03	−0.17
Education, years	−0.64	0.47
Low income	0.02	−0.03
Illness duration, years	0.25	−0.35
Psychotic disorder	0.16	−0.50
Day-program participation	0.16	0.68
Weekly family contact	0.03	0.45
